# Social function in adolescent eating disorders: lived experience framework for clinical practice

**DOI:** 10.1192/bjo.2025.10816

**Published:** 2025-09-22

**Authors:** Dasha Nicholls, Daniella Boules, Nikita Julius, Emerie Sheridan, Victoria Burmester

**Affiliations:** Division of Psychiatry, Department of Brain Sciences, Imperial College London, London, UK

**Keywords:** Anorexia nervosa, social functioning, child and adolescent psychiatry, feeding or eating disorders, rehabilitation and social psychiatry

## Abstract

**Background:**

Social function is increasingly demonstrated as a factor in risk, maintenance and outcome of eating disorders, but not emphasised in theoretical models of, and treatment approaches to, adolescent eating disorders.

**Aims:**

To adapt Schmidt and Treasure’s cognitive interpersonal model of anorexia nervosa to incorporate developmental and transdiagnostic components.

**Method:**

Qualitative interviews with young people aged 12–16 years (inclusive), who are in contact with child and adolescent community eating disorders services, and their parents, subjected to thematic analysis.

**Results:**

Five key themes emerged that were mutually dependent on a sixth theme of emotion regulation and coping. These themes were: peer relationships, change and uncertainty, thinking styles, appearance and achievement-based values, and family relationships.

**Conclusions:**

Peer relationships emerged as distinct from family relationships in this population, and a unifying theme was emotion regulation and coping. The framework could guide clinical assessment and the development or adaptation of interventions to address the themes identified. Research is needed to understand the role of the themes in treatment response and outcomes.

Social brain networks undergo dramatic changes during adolescence,^1^ resulting in heightened social awareness and self-consciousness, peer-directed social interactions and increased complexity of relationships.^2^ Early adolescence is associated with greater emotionality, and fosters abstract reasoning and social stress susceptibility.^3^ Peers surpass parents as the primary source of social support,^4^ and motivation for intimate interpersonal relationships increases.^5^ Peer relations have a protective effect against mental ill health,^6^ but social anxiety can disrupt their formation and stability. Abstract reasoning and emotional processing skills emerge, but inhibitory control over risky behaviours does not develop until later adolescence.^7^ Intact hormonal systems are necessary for these processes to develop, systems that are affected by illness. Adolescence is therefore a period of learning and brain growth, during which mental health interventions may have particular success.^8^

This sensitive period coincides with the onset of eating disorders. Eating disorders affect over 13% of young people and adult patients,^9,10^ with a cost to the UK economy of £15 billion/year.^11^ According to Beat, the UK eating disorder charity, eating disorder prevalence has increased 7% year on year since 2009,^11^ and hospital admissions for eating disorders have risen, although this trend may be changing.^12^ Despite their prevalence, our understanding and treatment repertoire is limited, and eating disorder research funding is inadequate. Social cognition and the quality of interpersonal relationships predict the development of eating disorder^13^ course and outcome.^13,14^ Up to 37% of adults with anorexia nervosa – the most common diagnosis in eating disorder services – have an autism spectrum disorder (ASD) or ASD features,^15^ with slightly lower estimates in adolescent eating disorder populations. Prolonged illness accentuates ASD traits, but ASD is sexually dimorphic, so, in the absence of a strongly suggestive developmental history, it is often difficult to diagnose in females who may have higher cognitive empathy. Since eating disorders disproportionately affect females, this may result in underdiagnosis.

Causal or maintenance models of eating disorders have largely been developed and empirically tested in adult populations. The cognitive interpersonal model of anorexia nervosa (Fig. 1), outlined initially in 2006, has underpinned studies examining cognitive and emotional traits in anorexia nervosa, and to develop and test interventions. Most recently, the model has been applied to the neuroprogression that results in chronic illness,^16^ and suggests that autism spectrum conditions (ASC) and traits have no direct impact on physical outcomes or eating disorder symptoms, but could be associated with higher rates of comorbidities and greater use of intensive eating disorder treatment. Patients with ASC characteristics may benefit more from individual rather than group sessions.^17^ Treatment adaptations such as the ‘PEACE’ pathway, a co-produced approach to anorexia nervosa comorbid with ASC,^18^ show early evidence of cost-savings and favourable treatment outcomes, suggesting that recognising and addressing aspects of social function within treatment approaches has the potential to influence course and outcome.

Application of the cognitive interpersonal model to young people has not been fully explored. One study that included young people found they had a better treatment response than adults to specialist in-patient or day patient treatment for anorexia nervosa (adult units *n* = 12; adolescent units *n* = 2; total patients *n* = 177), and that carer behaviour and interpersonal functioning may influence the response to in-patient care and recovery.^18^ Yet evidence that problems in social functioning are pertinent to the onset, course and outcome of eating disorders is mounting. Characterising social difficulties unique to young people with eating disorders, and understanding their bidirectional relationship to eating disorder symptoms and progression, is an important next step for potential identification of prodromal symptoms and the design of new interventions.

**Fig. 1:**
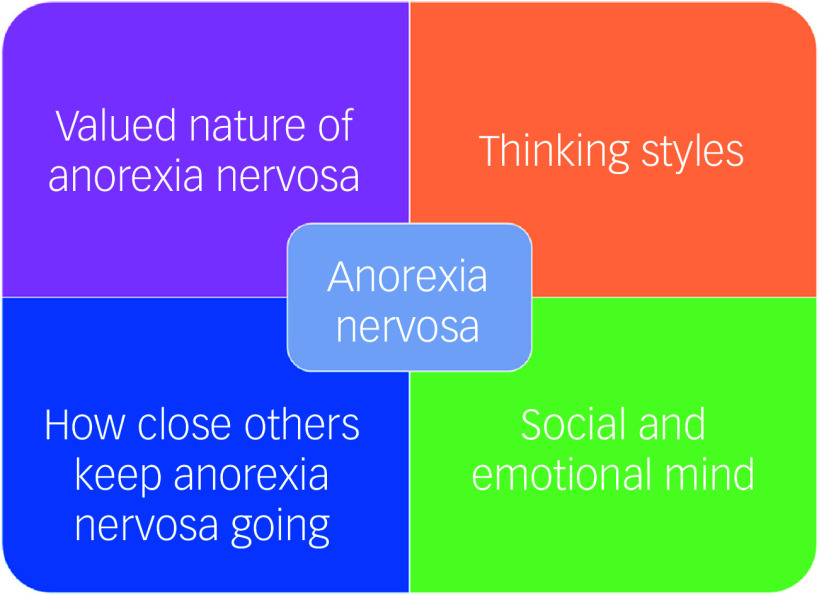
The cognitive interpersonal model for anorexia nervosa.^19^

The Medical Research Council framework for the development of complex interventions proposes theory development as a first step in the process of intervention development. Consulting young people and parents with lived experience of eating disorders when critiquing and adapting theoretical models ensures that science and research is addressing the needs and concerns of the group they intend to help. The aim of this study was to work with young people and their parents to integrate their views with contemporary scientific findings on social function in eating disorders into the cognitive interpersonal model, as a framework for psychological and pharmacological intervention in this population. The study was part of a programme of work examining the relationship between various aspects of social and interpersonal function in young people with eating disorders.

## Method

Adaptation of the model was informed by recent literature and qualitative data from repeated sessions with a young person and parent focus group run at Imperial College London, for people with experience of eating disorders with or without ASC. Participants were recruited via poster advertisements in four community eating disorder clinics within NHS Trusts treating young people with anorexia nervosa or bulimia nervosa. Participants completed an online screening form to ensure adherence with inclusion and exclusion criteria, and to provide informed consent or parental consent and young person assent to participate.

Inclusion criteria were as follows: (a) young people or parent of a young person aged 13–16 years, with anorexia nervosa or bulimia nervosa (bulimia nervosa), with or without comorbid ASC, treated at a community eating disorder service; (b) fluent in English and (c) have internet access.

Exclusion criteria were as follows: (a) presence of neurological pathology, (b) presence of other serious mental illnesses (e.g. schizophrenia, bipolar disorder), (c) significant life event in the past 30 days (such as a bereavement or financial windfall), (d) hearing or sight impaired without correction, (e) diagnosis of ASD (parent only) and (f) presence or history of eating disorder (parent only).

Objectives of the focus groups for this study (topic guide available from the authors) were to explore:

- What is the understanding of parents and young people with lived experience of anorexia nervosa/bulimia nervosa of the role of social functioning in the onset and maintenance of eating disorders?
- What model best captures the role of social and interpersonal functioning in the onset and maintenance of eating disorders in adolescents?
- How acceptable and valuable to parents and young people with lived experience of anorexia nervosa/bulimia nervosa is the development of new psychological and psychopharmacological interventions that target the social domain?

### Data analysis

Data from young people and parents generated were analysed using reflexive thematic analysis, by researchers experienced in social functioning and eating disorders (authors: V.B., N.J. and E.S.). After immersion in the data, each coder independently generated initial themes. We addressed reflexivity by critically examining our own influence on the research process, including our assumptions, positionality and interactions with participants. This involved group discussions to reflect on individual interpretations and potential biases, as well as documenting strong reactions and analytical decisions during manuscript review. To reduce bias, a fourth researcher (D.B.) drew the preliminary themes together from the coders for the parent group and the young person group, and consensus was achieved via meetings with the initial three authors who coded the scripts. A fifth author with expertise in young people with eating disorders (D.N.) synthesised the parent and young person themes and revised them, drawing on the literature. There was no difficulty in reaching consensus with initial codes and themes that were generated individually. No software was used to assist analysis.

## Results

Participants were two parent/child dyads: a 13-year-old child with ongoing severe anorexia nervosa, and a 15-year-old child with anorexia nervosa who was recovering. The latter also had a 19-year-old sister in hospital with anorexia nervosa. A further three parents contributed without their children’s involvement. They were the parents of a 14-year-old daughter recently diagnosed with anorexia nervosa, and of a 16-year-old girl with anorexia nervosa and autism, who was diagnosed age 10 years.

### Key themes

**Fig. 2:**
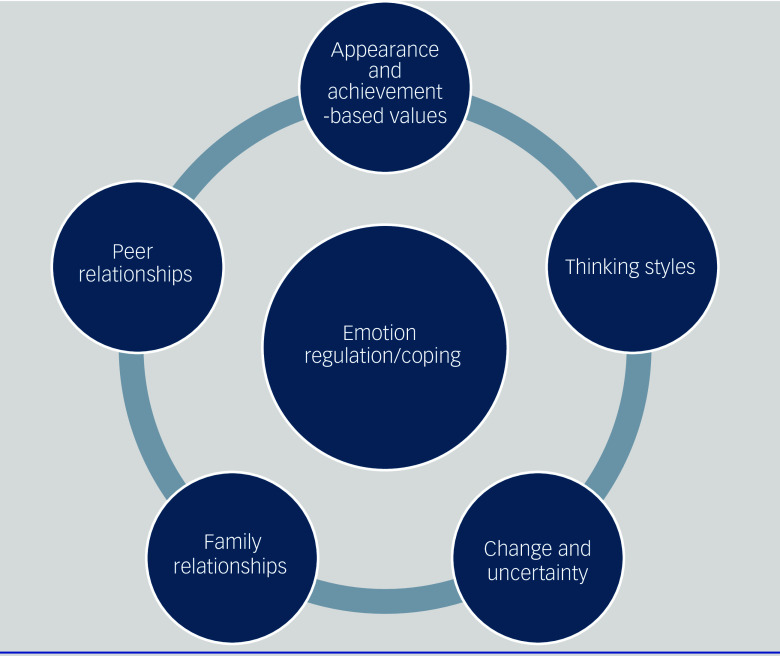
Developmental and transdiagnostic adaptation of the cognitive interpersonal model for adolescent eating disorders.

Five key themes emerged that were mutually dependent on a sixth theme of emotion regulation and coping (Fig. 2).

### Theme 1: peer relationships

**Key elements**: low empathy and low social connectedness.

Participants talked about having small friendship groups and the difficulty they had receiving and giving support to friends.

‘I certainly didn’t have a big group of friends and they’re not necessarily close.’ Young person

‘I have quite a small circle, probably about nearly four or five, and I wouldn’t say that I kind of talked to them in school, but not really out of school.’ Young person

‘I found it hard to trust them enough…’ Young person

‘I’m not very good when it comes to sort of people comforting each other.’ Young person

‘It just sort of made me come across as sort of not listening, thinking that they’re not important to me.’ Young person

‘I don’t I think that I’m generally there for my friends when they need me. But I don’t… that does not come from a place of dislike or annoyance or anything. It’s just from being busy.’ Young person

These challenges around friendships became more marked at the height of their illness, when they were focused on their own issues.

‘Maybe sometimes I’m a little bit distracted with myself and like school work. I know it sounds like really selfish.’ Young person

‘I think that because sometimes I can be so caught up in my own thoughts and like obviously cause… I’m just not sometimes not in the mood for putting on a brave face in front of them.’ Young person

‘I just feel so bad for my friends cause I was probably not a really nice person around that time. You know I wouldn’t have been able to support them if they wanted to talk to me about anything.’ Young person

These themes were echoed in parents’ accounts.

‘Now she has a very small group of friends… but I would say that she would find it very difficult to make new friends.’ Parent

‘And umm it was one of the really big things I think was ..sort of holding her back. I think she became – and she would agree that she became - not a very nice person to hang out with. Really. Withdrawn, moody.’ Parent

‘She really got used to having no friends and it didn’t seem to worry her. And we found that really sad as parents.’ Parent

‘It’s very difficult to separate the autism from the eating disorder for our daughter. Because that definitely affects her friendships in that she doesn’t see the need for people.’ Parent

Nonetheless, social support and connection was something both young people and parents viewed as important.

‘I basically missed all my friendships, broke down just because I did not have the energy to be speaking to anyone because I was, so I was struggling to keep myself alive.’ Young person

‘Now I try and make myself listen to music so I can talk to them about it and join in on their conversations.’ Young person

‘Umm, so now she’s got a she’s sort of found a really nice group of friends who are really supportive, and she feels really comfortable with.’ Parent

As well as not feeling they fitted in socially, being a victim of bullying featured strongly in young people’s narratives.

‘I know a lot of people just disliked me for some reason because I had many different interests and didn’t necessarily like what they did.’ Young person

‘I think when I was a lot heavier and I was not… I was bullied more… Even though I’m a pretty unpleasant person.’ Young person

‘She was targeted by one particular girl for the whole of year seven and the school were very much like, it’s not bullying. It’s just you, you know, parents dramatising kind of thing. Then she started self-harming, and they realised when we sent various screenshots and stuff….’ Parent

‘Another girl who was also unwell outed her private story, and that resulted in complete strangers calling her names, spitting at her, harassing her.’ Parent

### Theme 2: change and uncertainty

**Key elements**: pubertal change, school transition, adverse childhood experiences.

Although struggling with change may reflect a degree of cognitive inflexibility, childhood and adolescence is associated with changes outside of young people’s control. For example, pubertal changes brought increasing self-awareness, whereas external changes, such as change of school at age 11 (or 13) years, or resulting from the COVID-19 pandemic, rendered the young person’s world unpredictable. Change has potential to be accompanied by preoccupation with predictability and control. This might apply to friendships (small manageable groups of friends), food (eating at specific times, following dietary rules) or appearance (pigtails exactly symmetrical).

‘(Friends) not being, like, clear or precise with what they want … I’m a bit like overly specific about things, but just saying like I’ll be there and I’ll be there or giving me a time frame because I like plan my entire day around that one thing.’ Young person

‘I quite like things being planned out, so if the plan doesn’t go the way that I want it to, it can cause me a lot of stress and feel overwhelmed.’ Young person

‘Things being orderly and in a specific way and pattern is very reassuring and comforting.’ Young person

‘“A” used to be very popular and have a very large group of friends. Then they all went to one [secondary] school and she went to another and since then she’s never really been able to find her feet with the right group of people. And this is when all the problems started ….’ Parent

‘We’ve gone through a spate of cancer and lots of people and, you know, even their karate teacher died recently.’ Parent

‘Moving schools, COVID, the way this girl treated her – none of that was within her control.’ Parent

### Theme 3: thinking styles

**Key elements**: negative interpretation bias, rejection sensitivity, interpersonal threat perception, low self-worth, rigidity.

In addition to the cognitive inflexibility (difficulties with set-shifting) and perseveration (central coherence) that have been described in anorexia nervosa, a tendency to negative cognitive styles was noted across eating disorder diagnoses were evident in young people’s narratives, aligning with the literature. These include negative interpretation bias, sensitivity to social rejection and low self-worth, such that even pre-planned events perceived as exciting were disappointing when they happened.

‘I don’t want to look at myself and it causes me to feel very low and just like not in the mood to do anything or talk to anyone.’ Young person

‘Often because like I just overthink most of the things I say, I will be too embarrassed.’ Young person

‘if something embarrassing happened at school it would you put me off school for quite a while. I overthink it and too embarrassed for a while because I’m so like my ego is so bruised.’ Young person

‘I was so insecure, but I know that a lot of people treated me very differently from how they did when I lost weight and when I lost weight, people would often compliment me much more, even though I looked terrible, and I felt terrible and I was just a very unpleasant, unpleasant person to be around.’ Young person

Parents also identified sensitivity to social rejection as key triggers of their child’s distress, although interestingly, thought that having autism may provide some protection from this.

‘(Friends) would organise to meet up and then not tell her and then say, oh, you know, you can come at the last minute and then she’d go over and then they would not speak to her the whole evening, just completely blank her. I’d go and pick her up and she would be absolutely just in floods of tears.’ Parent

‘The more she sort of felt rejected, the more she felt she couldn’t speak to them… It’s sort of a kind of a cyclical thing.’ Parent

‘In a way, I’m glad that she’s autistic because I think she’s able to block that out more than maybe my other child who would find that a lot harder.’ Parent

They also mentioned the extent to which rigid thinking and need for control was a factor.

‘You know sort of timings and you know, only with that particular spoon or, you know, it has to be at 6:00 o’clock on the dot.’ Parent

‘My daughter will make an instant decision about a person – there is no changing her mind.’ Parent

‘I honestly don’t know anymore. I thought it was about being thin. Then I thought it was about control. Now I think it’s autism driven.’ Parent

### Theme 4: appearance and achievement-based values

**Key elements:** overvaluation of the thin ideal and perfectionism.

A strong narrative about desire for approval from others, especially friends, was evident, with an emphasis on both appearance and achievement. Academic achievement often took precedence over friendship. In addition to beliefs that people are treated differently on the basis of their weight and shape, young people talked about the importance of makeup, skincare and appearance more broadly, for example, changing behaviours to fit in with or impress peers, such as picking up mannerisms. They did note that close friends tended not to judge on the basis of appearance, while recognising that they treated people differently themselves on this basis.

‘I would in my mind I’d like, ohh, they’d think I’m really overweight. They think I’m fat. They all hate me.’ Young person

‘If I had to choose. I know I would pick the nicer looking one like to be friends with.’ Young person

‘The fact that I’ve been successful, and I have that validation from complete strangers that don’t know me is very reassuring.’ Young person

‘I hold myself to a really high moral standard, and if that’s being questioned by anyone… I have like basic core values that I live by.’ Young person

‘I think she sort of wondered with, you know, would people still like her if she wasn’t the sort of super super skinny person.’ Parent

‘I’ve definitely seen more perfectionism coming in and stupid little things. Like if her plaits aren’t symmetrical, she’ll have a meltdown… or eyelashes, ones got more mascara on than the other and that’s the end of the world. All these things that we would never have had before the anorexia.’ Parent

School was perceived as having an important role in driving perfectionism and competition rather than facilitating social connection, with a lot of importance placed on success and a highly negative opinions of failure.

‘I strive for success and I like, I like success. But I like to know that I succeed at everything. I can’t just go out and know that I’ve failed, that’s quite a mood kill for me.’ Young person

‘I think if I don’t succeed, I don’t feel as important to anyone.’ Young person

‘The fear that I feel is a bit irrational in that it’s like I’m scared that I’m gonna do something wrong even if I haven’t done it wrong yet.’ Young person

’She’s luckily very academic but she obviously feels she’s not good enough and that that breaks my heart more than anything, I think because she is perfect just the way she is.’ Parent

‘She’s just very sort of self-motivated and her friends don’t seem super motivated in the same way. So, it actually defines her.’ Parent

### Theme 5: family relationships

**Key elements**: suboptimal communication with parents, low parental self-efficacy, parent reflective function, familial affection and support.

The central role that parents/family play in young people’s eating disorder journey is reflected in the conflicted relationships with parents, particularly mothers, seen both as their biggest support/champion (major sacrifices made to be available to support, lots of gratitude) and as the enemy (making the young person eat, not trusted as motive is to get them to eat). Families were important for ensuring meals were eaten when possible, and despite being moody, rude and withdrawn during their illness, causing arguments at home, there were also opportunities for affection and confiding. A positive aspect was how their illness had enabled young people to strengthen their relationship with their parents and family.

‘It took me a while to open up to my mum, but after a while I did open up to my mum and dad about what was happening and then they went to the school because they thought that it obviously shouldn’t be happening.’ Young person

‘It took me a while to realise that they were there for me and it took me a while to start to open up to them, but I grew more safe as such around them. It became easier for me to open up and so now if, if I feel strong enough to share how I’m feeling with them, then I go straight to them straight away.’ Young person

‘I get from my parents is reassurance more than anything else. Just because I constantly worry that I’m not enough.’ Young person

‘I think I feel a little closer with my mum because I spent a lot of time with her while I was ill cause you know she has to be with me all the time, so I’d have to tell her lots of things.’ Young person

‘Her little sister was her rock… Yeah, she was literally at her side, you know.’ Parent

‘And my dad. That was a bit difficult because he didn’t really understand what was going on, so he’d just get angry at me all the time and I just felt like he was really disappointed in me for like not being able to do stuff.’ Young person

‘My older sister… she was also very ill at the same time as me. And we had an absolutely terrible relationship… we just argue and argue.’ Young person

‘My dad is not very emotionally available if that’s what you call it, but if he’s feeling upset, he’ll just kind of bust up and then it will come out in like a massive rage.’ Young person

‘And you know, it has brought us closer in a way.’ Parent

### Theme 6: overarching theme of emotion regulation/coping

Linking all the main themes were the coping strategies around emotions that the young people emphasised as being crucial to their recovery. They saw these as interacting with each of the other elements rather than as a separate theme. They talked about the ‘real me’, the ‘sick me’, and the newly therapised self. Emotion regulation strategies included avoiding confrontation (such as the reality of their illness), punishing the self and of being too depleted of energy to experience fear or embarrassment. Conversely, they talked about learning to express emotions.

‘Managing my emotions can feel quite overwhelming because obviously there’s a lot of them, so managing them can sometimes feel quite overwhelming.’ Young person

‘I find it difficult to regulate them (emotions) on a daily basis. So like how I’m feeling and be able to express that without shame. Is quite difficult, so I often just tend to repress them.’ Young person

‘I tried to just avoid the mirror in general… in the past and it lets me to feel more negatively about myself.’ Young person

’Sometimes I didn’t know how to react to affection that was being shown to me.’ Young person

‘I just went quiet and stopped talking and would never go to school anymore.’ Young person

‘I think fear stops me.’ Young person

Parents experience of their child’s emotions reflected the inaccessibility that accompanied this avoidance, and the central role of control over self and others.

‘I would say the way that she shows her upset is by restricting her food or I mean she won’t even drink water now. So food and water intake and previous to that it was by the self-harm. I would know she would be having a really tough day by finding like a sharpener in their pocket. That would be a way of her saying to me. Mum, I need help. But now there is no emotional contact really.’ Parent

‘And basically, you know, all the emotion comes out when you try and make them eat. And then the distress around that….’ Parent

‘Maybe she is a perfectionist. Maybe she has control. And this was in the easy way to put herself into control of maybe thoughts that were uncomfortable for her.‘ Parent

‘It’s a mixture of not being able to read emotions and desperately needing that approval.’ Parent

## Discussion

In this paper, we worked with young people and parents with lived experience of eating disorders to adapt Schmidt and Treasure’s cognitive interpersonal maintenance model of anorexia nervosa to young people and to a wider diagnostic profile, in line with emerging literature on social and emotional functioning in adolescent eating disorders. Where applicable, we retained language used in the Schmidt and Treasure model and, similarly, our adaptation aims to emphasise the interaction between vulnerability traits and eating disorder behaviours, and how they serve to maintain the disorder over time. Although some elements are similar, key differences related to biological and social transitions inherent in adolescent development emerged, and young people and parents suggested a role for some factors in onset as well as maintenance.

Peer relationships and the challenges of developing and maintaining friendships emerged as an area of importance for young people, and as quite distinct from family relationships. Social function is an important outcome predictor across mental disorders, and social cognition in childhood (as rated by parents when their child was age 7 years) strongly predicts disordered eating in later adolescence. This effect was significantly stronger for social cognition than for emotion recognition or bullying, and much stronger for disordered eating outcomes than for self-harm.^20^ Difficulties in social interaction may take the form of social anxiety, a risk factor for eating disorder; for others, social difficulties may be one element of broader deficits in social function, signalling possible ASC traits such as low empathy and poor social cognition. Research suggests a role for ASC and ASC traits as both a risk factor and prognostic indicator, although data are clearer for adults than for adolescents. Reliably diagnosing emerging neurodevelopmental disorders can be challenging when the emotional and cognitive deficits associated with eating disorders are dominant and autism assessments may not be readily available. Brief, objective and valid measures of neurocognitive processes that suggest ASC are needed to facilitate the diagnostic process, and clarify whether treatment adaptations, such as those outlined in the PEACE pathway, may be required.

Regardless of their nature, social difficulties and low levels of social connectedness rapidly become a perpetuating factor as the eating disorder isolates the individual from normal adolescent interactions, especially if prolonged hospital stay is required. Even when treated in the community, young people’s thinking styles, preoccupations and ‘reality testing’ can impair peer as well as family relationships. Steiger and colleagues demonstrated the prognostic significance of pretreatment social adaptation, defined as social and vocational adjustment, DSM-III-R Axis-V ratings (Highest Level of Adaptive Functioning Past Year on a scale of 0-100), and ‘object-relations’ capacities, to multimodal treatment for adults with bulimia nervosa (*n* = 44).^21^ Pretreatment social adjustment explained substantial and significant proportions of variance in posttreatment binge/purge symptoms, after accounting for eating disorder severity and concurrent psychiatric symptoms. More research is needed to explore the role of social function, including social interactions on social networks, in adolescent eating disorders, and whether targeted interventions are of benefit to those for whom it is a significant contributor to their presentation.

Closely related to social function is the theme of thinking styles and cognitive biases that influence interpersonal functioning. Young people with anorexia nervosa and bulimia nervosa experience heightened sensitivity to social rejection and a negative bias toward their social environment, yet research in young people remains scarce. Rowlands et al^22^ found that interpersonal sensitivity predicted eating disorder symptoms, which was partially mediated by negative interpretation bias towards social rejection, and poorer social relationships were associated with more severe eating disorder symptoms. It is not clear whether or to what extent the results could be explained by ASC symptoms, which were not assessed. The study also found no attentional social biases when using a dot-probe test experimental paradigm with negative and positive valenced faces. Similarly, a recent study found that adolescents with eating disorders have more negative self-attribution bias than controls, even when controlling for depression, and experimental studies suggest that young adults with both anorexia nervosa and bulimia nervosa are hypervigilant to social rejection and avoidant of social reward.^23–25^ More studies are needed to determine how negative verbal appraisal relates to clinical course.

Studies addressing cognitive biases associated with eating disorders are ongoing. Rowlands et al found that remotely delivered computerised cognitive bias modification (CBM) training reduced expectations of social rejection in 67 (all but one) female adolescents with eating disorders randomised to receive CBM in addition to treatment as usual (TAU).^26^ Young people who completed the intervention (22/37 randomised) displayed a significantly greater reduction in negative interpretations of ambiguous social scenarios, and eating disorder psychopathology compared with TAU, suggesting that CBM might be a useful treatment adjunct for some patients. There were no significant between-group differences on emotional responses to criticism, or on anxiety and depression symptoms.

Cognitive remediation therapy (CRT) is an adjunct treatment targeting set-shifting and weak central coherence, thought to play a maintaining role in anorexia nervosa, whereas cognitive remediation and emotional skills training aims to address cognitive and emotional factors.^27^ Nine studies included in a systematic review and meta-analysis of CRT in young people suggest improvements in cognitive performance with small effect sizes, positive patient feedback and low drop-out.^28,29^ CRT for anorexia nervosa can be delivered remotely, although the risk of disengagement is slightly higher.^30^ However, assessment of set-shifting and central coherence in routine clinical practice may present practical challenges. Furthermore, a recent study of adolescent in-patients with anorexia nervosa found set-shifting and central coherence improved over time irrespective of whether patients received CRT,^31^ underscoring the extent to which cognitive inefficiencies may reflect state rather than trait. Although most studies have been undertaken in anorexia nervosa, both set-shifting and central coherence are comparably impaired in people with bulimia nervosa, although not in binge eating disorder,^32^ justifying inclusion in our adapted model.

In our model, we included perfectionism as a value rather than as a thinking style, because of its close association with the extent to which striving for success, including around weight and shape, was framed by young people as core beliefs. Meta-cognitive training for eating disorders^33^ is designed to address cognitive flexibility and perfectionism, with corresponding effects on eating disorder psychopathology. In a feasibility and preliminary efficacy trial, 35 adolescent females were randomised to TAU plus meta-cognitive training for eating disorders, or TAU alone.^33^ The meta-cognitive training condition had low attrition (<15%) and was positively received. However, between-group differences favouring meta-cognitive training for eating disorders were not sustained at 3-month follow-up.

Although we recognise that problems coping with change is associated with some of the factors outlined above, we included change as a separate construct because change is faster in this developmental period than at any other stage, barring the neonatal period. These biological, psychological and social changes are, for the most part, completely outside of young people’s control. For example, ‘social emotions’ (guilt, shame, embarrassment, pride, contempt) are more sensitive to hormones than those processing basic emotions (fear, disgust, psychic pain), which are better correlated with age. The ability to ‘put yourself in another person’s shoes’ is therefore highly dependent on a functioning hypothalamic-pituitary-adrenal axis. Furthermore, the brain undergoes up to 50% synaptic pruning during adolescence,^34^ making it vulnerable to stress during this period. The centrality of ‘self-control’ has been debated across eating disorder literature for decades.^35^ Young people’s narratives suggest the need for control in the context of constant transitions and a specific developmental period is qualitatively different from the need for self-control in an otherwise ordered life.^36^

The role of family relationships to the course, outcome and effectiveness of intervention in young people with eating disorders is well established in both anorexia nervosa and bulimia nervosa.^37,38^ Our adapted model highlights family communication and emotional support from family members. Parental expressed emotion, even marginally elevated,^39^ is an established mediator of treatment response,^40^ and targeting parent critical comments and parental warmth show preliminary efficacy.^41^ Enhancing parental reflective function, through individual or group mentalisation-based therapy interventions, is another possible avenue for therapeutic innovation in adolescent eating disorders,^42^ given the impact on treatment response.^43^

The final theme is around emotion regulation or coping strategies. Core elements of emotion regulation involve thresholds for emotional triggers, which are strongly related to cognitive processes; recognition of emotions in oneself and others (alexithymia and empathy); moderating emotional intensity, including acceptance of negative emotions; distress tolerance and the ability to use adaptive strategies to modulate emotions/influence behaviours.^6,44–46^ Emotional regulation is gaining traction as a key modifiable transdiagnostic factor in a range of mental disorders, from addiction to autism to self-harm.^47^ In their systematic review, Jewell et al found that mentalising difficulties and eating disorder pathology were correlated in adolescent eating disorders, with particular emphasis on poor emotion recognition.^48^ Learning to recognise and manage emotions is a core task of adolescence, as well as of most forms of therapy. Females generally have superior cognitive theory of mind compared with males, although no gender difference in affective theory of mind has been established.

In conclusion, the cognitive interpersonal model of anorexia nervosa was adapted with young people and parents to incorporate developmental factors and to fit a broader diagnostic profile. It emphasises peer relationships as distinct from family relationships, and highlights the central and unifying theme of emotion regulation. It is intended as a framework to guide clinical assessment and inform new or adaptations of existing interventions to address the themes identified. Further research is needed to understand the role of each of the themes in determining onset, treatment response and outcome, and explore treatment approaches targeting social functioning, e.g. directed psychological interventions and pharmacological agents such as psychedelics. Whether the model is applicable to young people with avoidant/restrictive food intake disorder if overvalued ideas about weight/shape is excluded or modified needs further exploration.

## Data Availability

Data are available from the corresponding author on request.
